# The impact of surgery and age on mortality with primary trachea malignant tumors: a retrospective study based on propensity-score matching analysis

**DOI:** 10.1186/s13019-023-02340-z

**Published:** 2023-07-10

**Authors:** Chen Ping, Jia Liang, Zhi-Yuan Liu, Jiang He, Ji-Yao Zhou, Hao Cheng, Guang-Da Yuan

**Affiliations:** 1 Department of Thoracic Surgery, Suzhou Wuzhong People’s Hospital, Suzhou, Jiangsu Province 215128 P. R. China; 2 Department of Thoracic Surgery, The First Affiliated Hospital of Soochow University, Suzhou, Jiangsu Province 215006 P. R. China; 3 Department of Internal Medicine, Zhabei central hospital, Shanghai, 200070 P. R. China; 4 School of Medicine, School of Life Science and Technology, Shanghai Tenth People’s Hospital of Tongji University, Tongji University, Shanghai, 200072 P. R. China

**Keywords:** Primary trachea malignancy, Survival, Surgery, Subgroup analysis, Old patients

## Abstract

**Purpose:**

This study aimed to explore the survival significance of surgery and age on the prognosis of patients with primary trachea malignancies.

**Methods:**

The entire cohort of 637 patients with primary malignant trachea tumors was used to perform the main analyses. The data of those patients were from a public database. Overall survival (OS) curves were drawn by the Kaplan-Meier method and compared by the Log-rank test. The univariable and multivariable Cox regression analyses calculated the hazard ratio (HR) and 95% confidence interval (CI) for overall mortality. The propensity-score matching analysis was used to reduce the selection bias.

**Results:**

Age, surgery, histological type, N classification, M classification, marital status, and tumor grading were identified as independent prognostic factors after eliminating confounding factors. The results of the Kaplan-Meier method revealed that patients with age < 65 had a survival advantage over those with age ≥ 65 (HR = 1.908, 95% CI 1.549–2.348, *P* < 0.001). The 5-year OS rates were 28% and 8% in the group with age < 65 and age ≥ 65, respectively (*P* < 0.001). Cases with surgery had better survival over patients without surgery (HR = 0.372, 95% CI 0.265–0.522, *P* < 0.001). Compared with patients who did not undergo operations, patients with surgery had a higher median survival time (20 vs. 174 months). For patients with surgery, young age was considered a survival-promoting factor (HR 2.484; 95% CI 1.238–4.983, *P* = 0.010).

**Conclusion:**

We suggested that age and surgery were the independent prognostic factors in patients with primary malignant trachea tumors. Besides, age serves as an essential indicator for evaluating the prognosis of postoperative patients.

**Supplementary Information:**

The online version contains supplementary material available at 10.1186/s13019-023-02340-z.

## Introduction

The incidence rate of primary malignant tumors in the trachea is still rare and accounts for lower than 2% of all airway malignancies, according to previous reports [1, 2]. Besides, the symptoms of trachea malignancies are relatively subtle, and patients usually present with advanced disease when they are diagnosed definitely [3]. The approaches of treatment mainly include surgery, radiotherapy, and chemotherapy [3–6]. Surgery has been confirmed to be associated with survival advantages [5]. However, the important prognostic factors for patients after operation need to be further explored.

Age as a factor affecting the survival of other malignancies (except malignant tracheal tumors) has been investigated by many studies [7–9]. Researchers paid more attention to the effect of pathological features (such as complete resection, histological types, and grading), surgery, radiotherapy, and combined stage on the prognosis of patients with trachea malignancies [1, 3, 4, 6, 10]. However, the prognostic role of age in primary malignant trachea tumors, especially in cases with operations, is unclear. Therefore, this study aimed to uncover the survival significance of surgery in cohorts with different characteristics and the impact of age on the prognosis of patients with primary trachea malignancies.

## Methods

### Patients

**Fig. 1:**
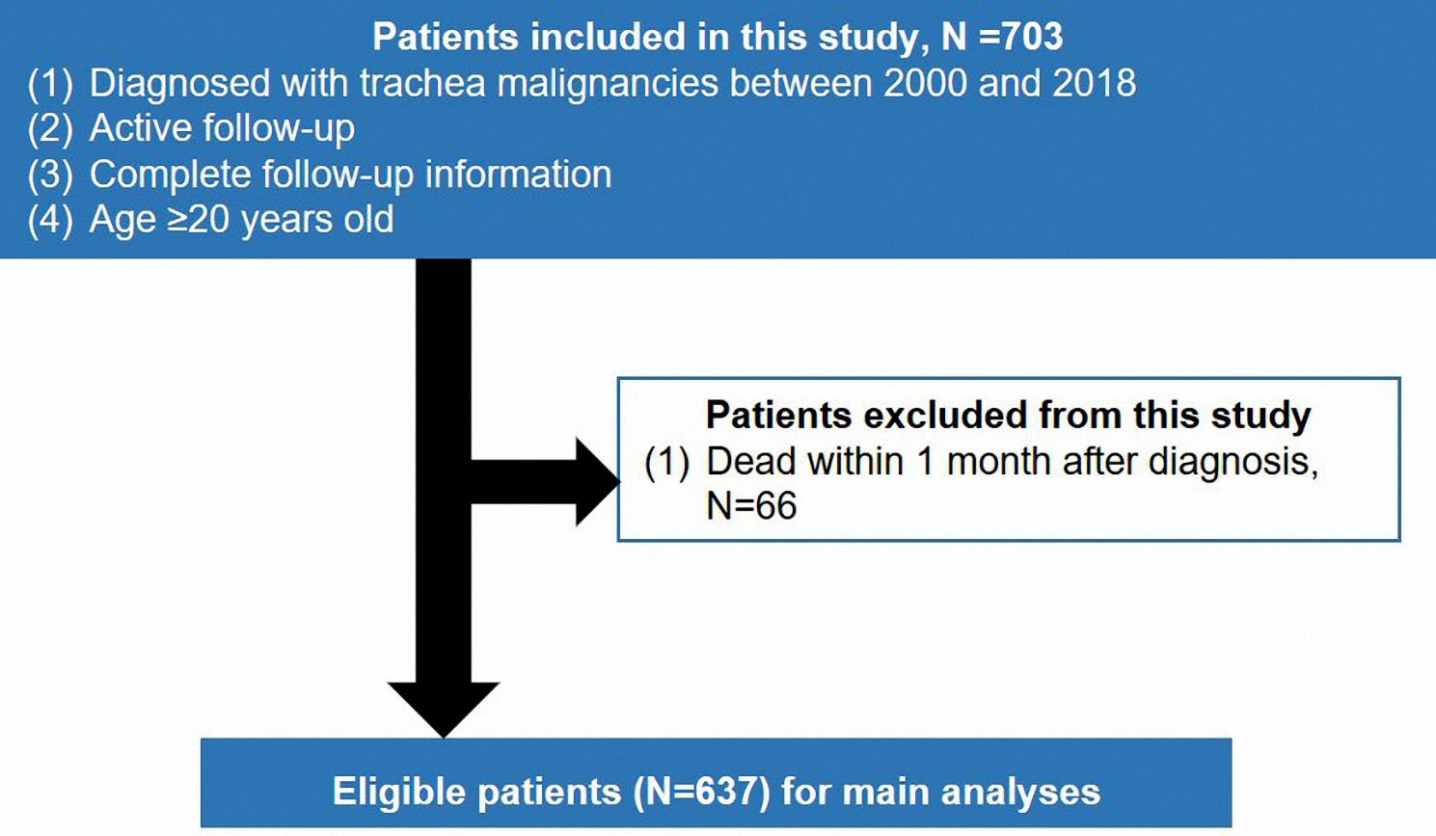
The flowchart of patient selection

The Ethics Committee of Suzhou Wuzhong People’s Hospital approved this study and considered this study exempt from ethical review. Patients were diagnosed as malignant tumors of the trachea in the Surveillance, Epidemiology, and End Results (SEER) database. All patient records were anonymized before analysis. The information about eligible patients was used to perform the main analyses. The inclusion criteria of patients were as follows: (1) diagnosed with primary trachea malignancies between 2000 and 2018; (2) age was over 19 years old; (3) active follow-up and complete follow-up information (N = 703). Patients who met the following standard were excluded from this study: (1) dead within one month (N = 66). Eventually, 637 patients were included in this study for main analyses. The flow chart is presented in Fig. 1. We collected information from the SEER database, including sex, race/ ethnicity, age, marital status, surgical treatment, radiotherapy, chemotherapy, tumor size, tumor grading, histological subtype, tumor extension, node involvement (N), metastasis describer (M), and follow-up duration as previous studies [11, 12]. Besides, we regarded 65 years as the cutoff value of age according to the published articles [11, 12]. We defined N0 as no lymph node involvement, N1 as lymph node involvement, Nx as unknown information about lymph node involvement, M0 as no distant lymph node or organ metastasis, M1 as distant lymph node metastasis, M2 as distant organ metastasis, and Mx as unknown information about distant metastasis because there was no standard staging system in primary trachea malignant tumors.

### Follow-up

The follow-up information was complete. Therefore, those patients had definitive survival statuses, including death and alive. Follow-up duration in cases ranged from 0 to 220.0 months, with a median of 22.0 months. Overall survival (OS) was the duration from the date of diagnosis to death. We considered OS as our primary observational endpoint. Since detailed information on follow-up in the SEER database is not clear, we recommend that patients return every 3–6 months for the first two years after surgery and every 12 months starting in the third year after surgery for chest and neck computed tomography and tumor biomarkers, tracheoscopy if necessary and positron emission tomography-computed tomography according to our experience.

### Classification of tumor extension

According to the guidelines from SEER (https://web2.facs.org/cstage0205/trachea/Trachea_bcf.html), we classified the statuses of tumor extension into five types, including E1 (tumor was confined to the trachea), E2 (tumor spread outside the trachea but not to adjacent connective tissue), E3 (tumor spread to adjacent connective tissue, organs or other structures), E4 (further contiguous extension), and Ex (unknown extension).

### Statistical analysis

All statistical analyses were performed using SPSS Statistics 25.0 software (IBM SPSS, Inc., Armonk, IL, USA). The hazard ratios (HRs) and 95% confidence intervals (CIs) were calculated using univariable and multivariable Cox regression analyses (the regression method was Enter selection). The survival curves were drawn by the Kaplan-Meier method and compared by the Log-rank test. Statistical tests were considered statistically significant with a two-sided *P* value < 0.05. Propensity-score matching (PSM) analysis with a ratio of 1:1 was performed to reduce the selection bias. The match tolerance was 0.01. The Chi-square test was conducted to evaluate the equilibrium between groups.

## Results

### Patient characteristics

Table 1 presents the baseline characteristics of the entire cohort. In this study, men patients outnumbered women patients. Patients with age < 65 outnumbered those aged ≥ 65, constituting 50.1% of the patients. A total of 127 (19.9%) patients received operations, whereas 474 (74.4%) did not undergo surgery. The majority of patients were diagnosed with squamous cell carcinoma (SCC), comprising more than 52% of the patients. In terms of the tumor extension, most patients lost detailed information and were classified as Ex. The proportion of patients who underwent radiotherapy was high, reaching 64.5%. Besides, the characteristics before and after PSM are shown in **Supplementary Tables 1–2**.

**Table 1:** Clinical and pathological characteristics of patients with primary trachea tumors

Variables		N	%
Race	Caucasians	500	78.5
	Other	137	21.5
Sex	Male	371	58.2
	Female	266	41.8
Age (year)	< 65	319	50.1
	≥ 65	318	49.9
Marital status	Unmarried	250	39.2
	Married	345	54.2
	Unknown	42	6.6
Surgery	No	474	74.4
	Yes	127	19.9
	Unknown/other	36	5.7
Radiotherapy	No	211	33.1
	Yes	411	64.5
	Unknown	15	2.4
Chemotherapy	No	409	64.2
	Yes	228	35.8
Grade	Well-moderate	182	28.6
	Poor-undifferentiated	153	24.0
	Unknown/other	302	47.4
Extension	E1	158	24.8
	E2	69	10.8
	E3	143	22.4
	E4	13	2.0
	Ex	254	40.0
N classification	N0	267	41.9
	N1	88	13.8
	Nx	282	44.3
M classification	M0	332	52.1
	M1	5	0.8
	M2	42	6.6
	Mx	258	40.5
Histology	SCC	337	52.9
	SGC	136	21.4
	Other/unknown	164	25.7
Tumor size	≤ 3.0 cm	148	23.2
	3.0-5.0 cm	73	11.5
	> 5.0 cm	23	3.6
	unknown	393	61.7

SCC: squamous cell carcinoma, SGC: salivary-gland type carcinoma

### Univariable and multivariable analyses for the entire cohort

The outcomes of univariable and multivariable analyses for the entire cohort are presented in Table 2. Marital status, age, tumor extension, tumor size, N classification, M classification, surgery, tumor grade, and histological type were considered to affect survival after univariable analysis. Other variables, including race, chemotherapy, and radiotherapy, had no significant influence on survival in the univariable analysis. Besides, the multivariable analysis confirmed age, surgery, histological type, N classification, M classification, marital status, and tumor grading as independent prognostic factors after eliminating confounding factors.

**Table 2:** Univariable and multivariable Cox proportional hazard regression analyses for mortality in trachea tumor patients

	Univariable analysis		Multivariable analysis		
Variables	HR	*P*-Value	HR	95% CI	*P*-Value
Race/ ethnicity					
Caucasians	1				
Others	0.926	0.515			
Sex					
Male	1		1	reference	
Female	0.813	0.036	0.885	0.721–1.086	0.242
Marital status					
Non-married	1		1	reference	
Married	0.707	0.001	0.742	0.603–0.913	0.005
Unknown	0.923	0.684	0.847	0.568–1.264	0.417
Age (year)					
< 65	1		1	reference	
≥ 65	2.103	< 0.001	1.905	1.548–2.344	< 0.001
Surgery					
No	1		1	reference	
Yes	0.276	< 0.001	0.371	0.265–0.520	< 0.001
Other/ unknown	0.881	0.540	1.076	0.707–1.638	0.733
Chemotherapy					
No	1		1	reference	
Yes	1.392	0.001	0.845	0.683–1.045	0.120
Radiotherapy					
No	1				
Yes	0.943	0.576			
Unknown	0.831	0.590			
Histological types					
SCC	1		1	reference	
SGC	0.260	< 0.001	0.396	0.286–0.550	< 0.001
Other/unknown	0.644	< 0.001	0.783	0.616–0.997	0.047
Extension					
E1	1				
E2	1.145	0.457	0.769	0.528–1.120	0.171
E3	1.504	0.004	1.213	0.881–1.671	0.237
E4	2.565	0.002	1.403	0.718–2.740	0.322
Ex	1.456	0.004	1.546	1.064–2.247	0.022
N classification					
N0	1		1	reference	
N1	2.330	< 0.001	1.426	1.046–1.944	0.025
Nx	1.376	0.004	2.185	1.364–3.502	0.001
M classification					
M0	1		1	reference	
M1	2.648	0.032	1.238	0.492–3.112	0.651
M2	3.129	< 0.001	2.055	1.424–2.967	< 0.001
Mx	1.087	0.430	0.342	0.205–0.568	< 0.001
Grade					
I-II	1		1	reference	
III-IV	1.526	0.001	1.337	1.029–1.737	0.030
Unknown/other	1.005	0.966	1.068	0.837–1.363	0.596
Tumor size					
< 3.1 cm	1		1	reference	
3.1-5.0 cm	1.467	0.030	0.979	0.679–1.412	0.909
> 5.0 cm	2.087	0.004	1.570	0.903–2.731	0.110
Unknown	1.704	< 0.001	1.258	0.925–1.711	0.144

h: hazard ratio, CI: confidence interval, SCC: squamous cell carcinoma, SGC: salivary gland-type carcinoma

Cox regression’s method was the Enter selection

### Survival analyses

**Fig. 2:**
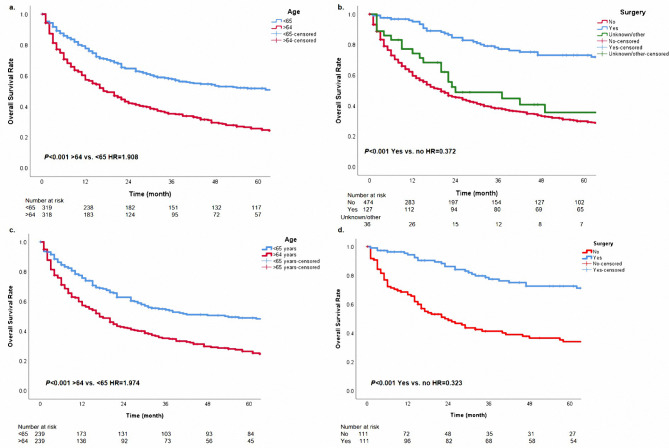
The overall survival curves based on age **(a)** and surgery **(b)**. The overall survival curves based on age (c) and surgery (d) after propensity-score matching

There were 426 death events (66.9%) in the cohort. The 1-year, 3-year, and 5-year OS rates were 70%, 42%, and 18% in the entire cohort, respectively. The median survival time for the entire cohort was 22.0 months. The results of the Kaplan-Meier method revealed that patients with age < 65 had a survival advantage over those with age ≥ 65 (Fig. 2a, adjusted HR = 1.905, 95% CI 1.548–2.344, *P* < 0.001). The 5-year OS rates were 28% and 8% in the group with age < 65 and age ≥ 65, respectively. Cases with surgery had better survival over those without surgery (Fig. 2b, adjusted HR = 0.371, 95% CI 0.265–0.520, *P* < 0.001). Compared with patients who did not undergo operations, patients with surgery had a higher median survival time (20 vs. 174 months). After PSM, surgery and age < 65 were also confirmed as protective factors (Fig. 2c-d, all *P* < 0.05).

### Sub-group analyses

**Fig. 3:**
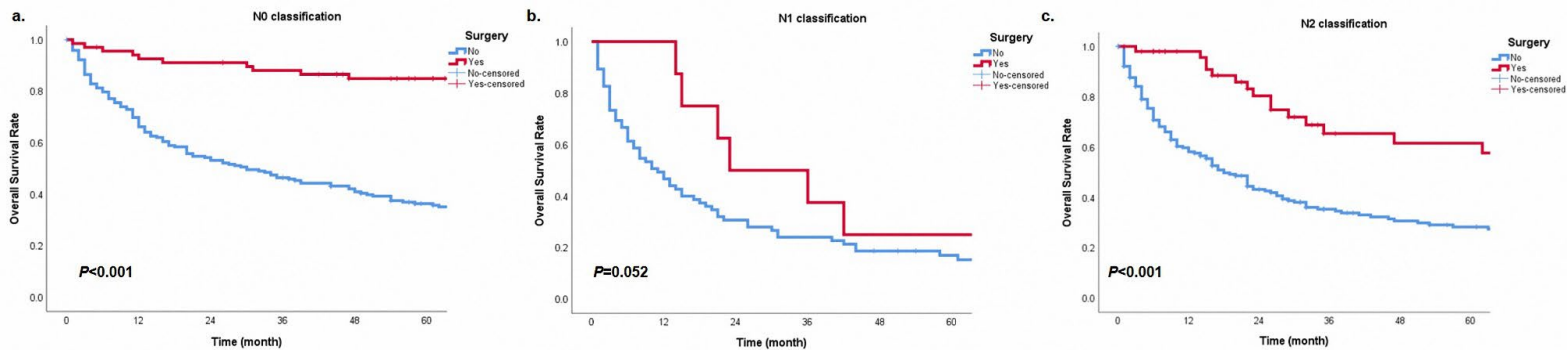
The overall survival curves based on surgery in different cohorts with classifications N0 **(a)**, N1 **(b)**, and N2 **(c)**

**Fig. 4:**
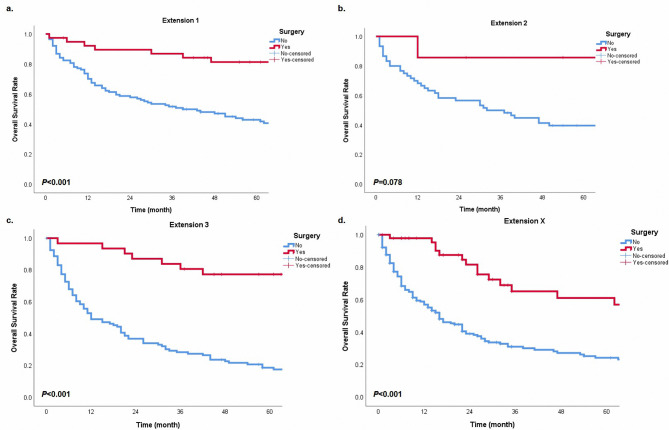
The overall survival curves based on surgery in different cohorts with extension 1 **(a)**, extension 2 **(b)**, extension 3 **(c)**, and extension x **(d)**

**Fig. 5:**
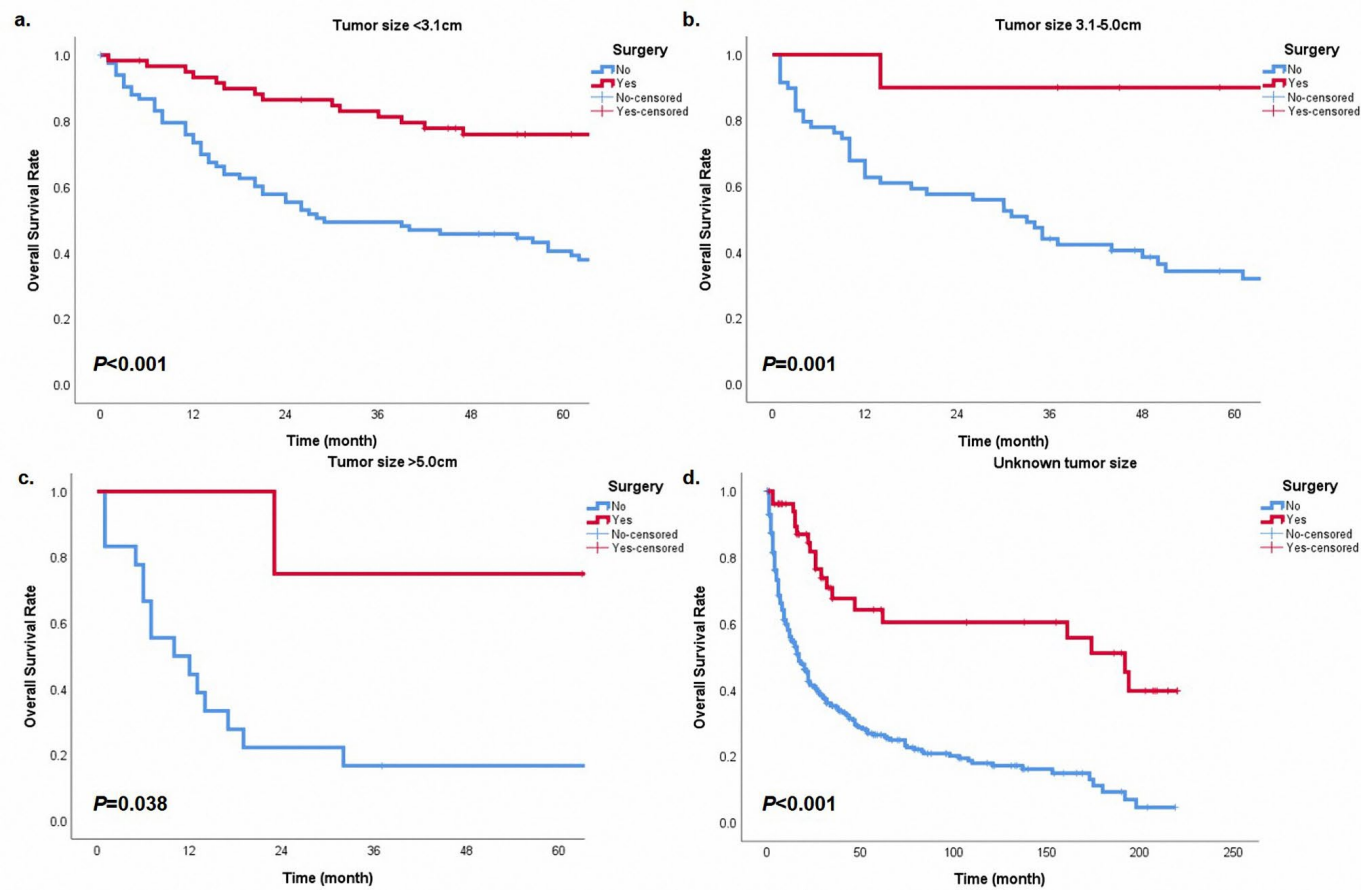
The overall survival curves based on surgery in different cohorts with tumor size < 3.1 cm **(a)**, tumor size 3.1-5.0 cm **(b)**, tumor size > 5.0 cm **(c)**, and unknown tumor size **(d)**

**Fig. 6:**
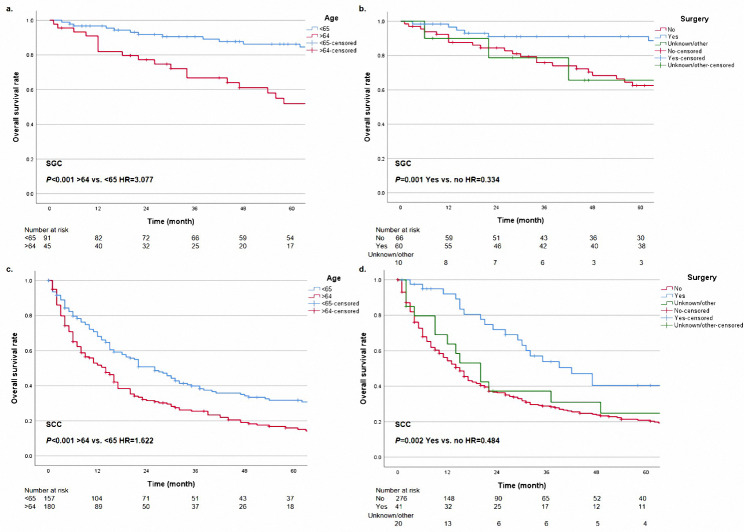
The overall survival curves based on age **(a and c)** and surgery **(b and d)** in different histological types

**Fig. 7:**
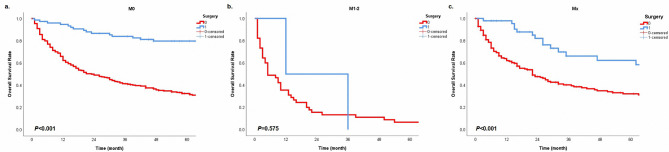
The overall survival curves based on surgery in different cohorts with classifications M0 **(a)**, M1-2 **(b)**, and Mx **(c)**

In order to further explore the prognostic significance of surgery in different subgroups, we performed the Kaplan-Meier method in cohorts with different characteristics. In groups with N0 or N2 classification, surgery provided patients with survival benefits (Fig. 3, all *P* < 0.05). Patients with E1, E3, and Ex could get survival advantages from operations (Fig. 4, all *P* < 0.05). However, in the cohort with N1 classification or E2, surgery did not have statistical significance in the prognostic benefit compared with non-surgery (Fig. 3b, P = 0.052; Fig. 4b, P = 0.078). Patients with surgery presented a better prognostic trend than patients without operations. Besides, cases could get a prolonged survival from surgery in any tumor size (Fig. 5, all *P* < 0.05). For different histological types, age and surgery could stratify patient prognosis of SGC and SCC (Fig. 6, all *P* < 0.05). Moreover, we performed analysis to explore the surgical significance of prognosis in patients with different M classifications (Fig. 7). We found that patients with surgery had more improved outcomes than patients without operations in cohorts with M0 or Mx classifications (all *P* < 0.001). Although the stratification curves did not demonstrate statistical differences in this group of patients (classification M1-2), the operated patients demonstrated a good prognostic trend (Fig. 7b).

### Multivariable analysis for the cohort with surgery

The multivariable analysis further identified the prognostic factors affecting survival in the cohort with operations. For patients with surgery, young age was considered a survival-promoting factor (HR 2.237; 95% CI 1.159–4.321, *P* = 0.010; Table 3). The 1-, 3- and 5-year OS rates of patients were 88.0% vs. 77.0%, 81.0% vs. 57.0%, and 52.0% vs. 29.0% in the group with age < 65 or > 64, respectively. Besides, histological type, tumor extension, and M classification also served as independent prognostic factors (Table 3).

**Table 3:** Multivariable Cox proportional hazard regression analyses for mortality in primary trachea tumor patients with surgery

	Multivariable analysis		
Variables	HR	95% CI	*P*-Value
Age (year)			
< 65	1	reference	
≥ 65	2.237	1.159–4.321	0.016
Chemotherapy			
No	1	reference	
Yes	1.526	0.558–4.174	0.410
Histological types			
SCC	1	reference	
SGC	0.296	0.144–0.608	0.001
Other/unknown	0.208	0.077–0.558	0.002
Extension			
E1	1	reference	
E2	0.955	0.184–4.966	0.956
E3	0.648	0.209–2.013	0.453
E4	6.174	1.064–35.82	0.042
Ex	1.351	0.101–18.07	0.820
N classification			
N0	1	reference	
N1	3.517	0.769–16.09	0.105
Nx	0.261	0.024–2.888	0.273
M classification			
M0	1	reference	
M1	9.210	1.485–57.13	0.017
M2	4.155	0.551–31.35	0.167

h: hazard ratio, CI: confidence interval, SCC: squamous cell carcinoma, SGC: salivary gland-type carcinoma

Cox regression’s method was the Enter selection. Sex, marital status, radiotherapy, tumor size, and grade did not enter into the multivariable regression because the two-sided *P* values were over 0.05

## Discussion

In the current study, we analyzed data on primary tracheal malignancies in the SEER database. The tracheal tumor is a rare malignant disease for which surgery is often the primary treatment. In this study, we verified and revealed the significance of surgery in primary malignant trachea tumors. Finally, we found that surgery did significantly improve survival for patients with tracheal tumors. In our subgroup analysis, we revealed that in different N classifications, tumor extension, and tumor size, the patients with surgery were more likely to obtain survival benefits than the patients without surgery. In addition, we uncovered that age could also be used as an independent prognostic factor to predict the prognosis of patients with tracheal malignancies. Younger patients had a better prognosis than older patients. To further explore prognostic characteristics in the surgical population, we performed a multivariable Cox regression analysis on patients undergoing surgery for tracheal tumors and found that age could still be an independent prognostic factor. When performing subgroup analysis, we did not perform the Kaplan-Meier analysis for the E4 subgroup of patients because there were too few patients with further contiguous extension (E4). Although in the subgroups with lymph node involvement (N1), distant lymph node metastasis (M1), distant organ metastasis (M2), and tumor spread outside the trachea but not to adjacent connective tissue (E2), after the Kaplan-Meier analysis, the *P* values ​​were all greater than 0.05, indicating no statistical significance, the survival curves showed a trend that surgical patients had a better prognosis than non-surgical patients. Our study thus confirmed that age and surgery could be prognostic factors in patients with malignant trachea tumors. For patients with primary trachea malignancies, we propose that surgery be performed as soon as possible after excluding surgical contraindications, and age is a factor that cannot be ignored in the process of evaluating the prognosis of primary malignant tracheal tumors.

Previous studies have explored the prognostic effect of age on patients with malignant trachea tumors [10, 13, 14]. However, the results were controversial. A study by *He J et al.* found that age as a continuous variable was considered an independent prognostic indicator [14]. They used the SEER database and analyzed the data of 287 patients. Interestingly, another study by *Wo Y et al.* revealed that age did not have a significant impact on the survival of patients with trachea malignancies [10]. *Wo Y* and his collogues also used data from the SEER database as *He J et al.*. The difference between the abovementioned studies was the processing of age variable. One of the studies put age directly into Cox regression as a continuous variable; however, the other put age as a categorical variable in Cox regression. *Mallick S* and collogues collected the case reports from online articles and analyzed the data of 733 patients [13]. They found that patients with age < 50 had better survival than patients with age > 49. Our study had similar results to theirs, indicating that patients with age ≥ 65 had a poorer prognosis over those with age < 65. Furthermore, we explored the reason why older age had poorer survival and found that old patients had a higher proportion of squamous cell carcinoma than younger patients. In the present study, patients with squamous cell carcinoma had the worst prognosis than patients with other histological types. In addition, investigated the prognostic impact of age on patients with malignant trachea tumors after resection and reached similar findings. Therefore, we suggest that age could serve as an independent prognostic factor for trachea malignancies patients. Older postoperative patients may need to find more appropriate follow-up and postoperative adjuvant treatment strategies.

The primary treatment approach for patients with primary malignant trachea tumors is still resection. Some studies confirmed the vital role of surgery on patients with trachea malignancies [13, 15, 16]. The findings of the present study validated that surgery could prolong the survival of patients with trachea malignancies. Besides, we further performed the sub-group analysis and found that in different N classifications, tumor extension, and tumor size, the patients with surgery were more likely to obtain survival benefits than the patients without surgery. A study by *Wo Y et al.* found that the lymph node ratio was an independent indicator in patients with surgery [10]. The cutoff value of the lymph node ratio was 0.07. In fact, a lymph node ratio of 0.07 was a low level, in other words, indicating one positive lymph node in all examined lymph nodes. Therefore, their results suggested that patients with metastasis of lymph nodes had decreased survival than those without lymph node metastasis. In the present study, we uncovered that the lymph node involvement (N1 classification) had poorer survival than the no lymph node involvement (N0 classification). Thus, it is crucial to perform the dissection of the lymph nodes and confirm the status of the lymph nodes.

The effect of chemotherapy and radiotherapy on survival of postoperative patients needs further to be explored. In the present study, we found that radiotherapy and chemotherapy could not provide survival benefit for postoperative patients. However, the proportion of postoperative patients receiving chemotherapy was low, only 15.0%. Therefore, the positive significance of chemotherapy might not be showed in the present study. Besides, the postoperative radiotherapy usually plays a compensatory role of operation. In the clinical practice, it’s important to perform radiotherapy for patients without complete resection (R0) [17, 18]. Thus, it is impossible to generalize whether postoperative radiotherapy can provide a survival benefit to patients undergoing surgery. We must first know whether the patient is a complete resection. Unfortunately, such data are missing inside the SEER database.

The current study still has some flaws. First, despite the large sample size of the SEER database, specific information on complete resection, perioperative complications, and tumor location is lacking. In fact, these results may have an impact on the findings of the study. Second, since this study was a retrospective database study, selection bias was unavoidable. Thus, we performed PSM to reduce the selection bias. In addition, more samples need to be collected for research. Third, since age has become a categorical variable in the database, we had to select patients over 19 years old to ensure that all patients were adults. In fact, in the categorical variable, the interval in which 18 years of age was located in group 15–19 years, which included a portion of patients who were underage. This left us missing information on patients in the 18–19 age group. Therefore, we need more studies to validate our results.

## Conclusions

We suggested that age and surgery were the independent prognostic factors in patients with primary malignant trachea tumors. Besides, age serves as an essential indicator for evaluating the prognosis of postoperative patients.

## Electronic supplementary material

Below is the link to the electronic supplementary material.

Additional File 1: The clinical and pathological characteristics before and after PSM according to the age group.

Additional File 2: The clinical and pathological characteristics before and after PSM according to the surgery group.

## Data Availability

Any researchers interested in this study could contact us to request the data.

## Abbreviations

SEER: Surveillance Epidemiology and End Results
N: node
M: metastasis
OS: overall survival
HR: hazard ratios
CI: confidence intervals
SCC: squamous cell carcinoma
SGC: salivary-gland type carcinoma
PSM: propensity-score matching

## References

[1] Bhattacharyya N (2004). Contemporary staging and prognosis for primary tracheal malignancies: a population-based analysis. Otolaryngol Head Neck Surg.

[2] Schneider P, Schirren J, Muley T, Vogt-Moykopf I (2001). Primary tracheal tumors: experience with 14 resected patients. Eur J Cardiothorac Surg.

[3] Xie L, Fan M, Sheets NC, Chen RC, Jiang GL, Marks LB (2012). The use of radiation therapy appears to improve outcome in patients with malignant primary tracheal tumors: a SEER-based analysis. Int J Radiat Oncol Biol Phys.

[4] Hogerle BA, Lasitschka F, Muley T, Bougatf N, Herfarth K, Adeberg S, Eichhorn M, Debus J, Winter H, Rieken S (2019). Primary adenoid cystic carcinoma of the trachea: clinical outcome of 38 patients after interdisciplinary treatment in a single institution. Radiat Oncol.

[5] Gaissert HA, Grillo HC, Shadmehr MB, Wright CD, Gokhale M, Wain JC, Mathisen DJ (2004). Long-term survival after resection of primary adenoid cystic and squamous cell carcinoma of the trachea and carina. Ann Thorac Surg.

[6] Liu XY, Liu FY, Wang Z, Chen G (2009). Management and surgical resection for tumors of the trachea and carina: experience with 32 patients. World J Surg.

[7] Wu L-L, Zhong J-D, Zhu J-L, Kang L, Huang Y-Y, Lin P, Long H, Zhang L-J, Ma Q-L, Qiu L-H et al. Postoperative survival effect of the number of examined lymph nodes on esophageal squamous cell carcinoma with pathological stage T1–3N0M0. BMC Cancer 2022, 22(1). 10.1186/s12885-022-09207-x PMC8800278 35090428

[8] Qian JY, Hao Y, Yu HH, Wu LL, Liu ZY, Peng Q, Li ZX, Li K, Liu Y, Wang RR, Xie D (2023). A novel systematic oxidative stress score predicts the survival of patients with early-stage lung adenocarcinoma. Cancers (Basel).

[9] Jiang WM, Wu LL, Wei HY, Ma QL, Zhang Q (2021). A parsimonious Prognostic Model and Heat Map for Predicting Survival following adjuvant Radiotherapy in Parotid Gland Carcinoma with Lymph Node Metastasis. Technol Cancer Res Treat.

[10] Wo Y, Li S, Wang Y, Lu T, Qin Y, Sun X, Jiao W (2018). Predictors of nodal metastasis and prognostic significance of lymph node ratio and total lymph node count in tracheobronchial adenoid cystic carcinoma. Cancer Manag Res.

[11] Liu YH, Wu LL, Qian JY, Li ZX, Shi MX, Wang ZR, Xie LY, Liu Y, Xie D, Cao WJ (2023). A Nomogram based on Atelectasis/Obstructive pneumonitis could predict the metastasis of Lymph Nodes and postoperative survival of pathological N0 classification in non-small cell Lung Cancer Patients. Biomedicines.

[12] Wu LL, Li CW, Lin WK, Qiu LH, Xie D (2021). Incidence and survival analyses for occult lung cancer between 2004 and 2015: a population-based study. BMC Cancer.

[13] Mallick S, Benson R, Giridhar P, Rajan Singh A, Rath GK (2019). Demography, patterns of care and survival outcomes in patients with malignant tumors of trachea: a systematic review and individual patient data analysis of 733 patients. Lung Cancer.

[14] He J, Shen J, Huang J, Dai C, Liang W, Ye M, Kong M, Chen B, Zhu C, He J (2017). Prognosis of primary tracheal tumor: a population-based analysis. J Surg Oncol.

[15] Mathisen DJ. 50th Anniversary Landmark Commentary on Grillo HC. Tracheal Tumors: Surgical Management. Ann Thorac Surg 1978;26:112 – 25. *Ann Thorac Surg* 2015, 100(1):6–7. 10.1016/j.athoracsur.2015.05.049 26140755

[16] Perelman M, Korolyova N (1968). Surgery of tumours in the thoracic portion of the trachea. Thorax.

[17] Predina J, Suliman R, Potter AL, Panda N, Diao K, Lanuti M, Muniappan A, Jeffrey Yang CF. Postoperative radiotherapy with modern techniques does not improve survival for operable stage IIIA-N2 non-small cell lung cancer. J Thorac Cardiovasc Surg 2022, 18:S0022-5223(22)01134-5. 10.1016/j.jtcvs.2022.09.062 36610886

[18] Gao Y, Fan X, Hua C, Zheng H, Cui Y, Li Y, Wu K (2023). Failure patterns for thymic carcinoma with completed resection and postoperative radiotherapy. Radiother Oncol.

