# Supplementary material for: The impact of surgery and age on mortality with primary trachea malignant tumors: a retrospective study based on propensity-score matching analysis
Source: J Cardiothorac Surg. 2023 Jul 10;18:224. doi: 10.1186/s13019-023-02340-z (PMC10334644; doi:10.1186/s13019-023-02340-z)
Supplement: Supplementary file 1 — Additional File 1: The clinical and pathological characteristics before and after PSM according to the age group. [file 13019_2023_2340_MOESM1_ESM.docx]

**Supplementary table 1**: The clinical and pathological characteristics before and after PSM according to the age group.

|  | | **Age** | |  | **Age (PSM)** | |  |
| --- | --- | --- | --- | --- | --- | --- | --- |
| **Variables** | **<65 years** | | **>64 years** | ***P* value** | **<65 years** | **>64 years** | ***P* value** |
|  | **N=319** | | **N=318** |  | **N=239** | **N=239** |  |
| Race |  | |  | 0.017 |  |  | 0.407 |
| Caucasians | 238 (74.6%) | | 262 (82.4%) |  | 199 (83.3%) | 192 (80.3%) |  |
| Other | 81 (25.4%) | | 56 (17.6%) |  | 40 (16.7%) | 47 (19.7%) |  |
| Sex |  | |  | 0.158 |  |  | 0.925 |
| Male | 177 (55.5%) | | 194 (61.0%) |  | 146 (61.1%) | 145 (60.7%) |  |
| Female | 142 (44.5%) | | 124 (39.0%) |  | 93 (38.9%) | 94 (39.3%) |  |
| Surgery |  | |  | <0.001 |  |  | 0.137 |
| No | 212 (66.5%) | | 262 (82.4%) |  | 177 (74.1%) | 189 (79.1%) |  |
| Yes | 84 (26.3%) | | 43 (13.5%) |  | 54 (22.6%) | 38 (15.9%) |  |
| Unknown | 23 (7.2%) | | 13 (4.1%) |  | 8 (3.3%) | 12 (5.0%) |  |
| Chemotherapy |  | |  | 0.260 |  |  | 0.850 |
| No | 198 (62.1%) | | 211 (66.4%) |  | 148 (61.9%) | 150 (62.8%) |  |
| Yes | 121 (37.9%) | | 107 (33.6%) |  | 91 (38.1%) | 89 (37.2%) |  |
| Radiotherapy |  | |  | 0.041 |  |  | 0.601 |
| No | 92 (28.8%) | | 119 (37.4%) |  | 82 (34.3%) | 72 (30.1%) |  |
| Yes | 217 (68.0%) | | 194 (61.0%) |  | 153 (64.0%) | 162 (67.8%) |  |
| Unknown | 10 (3.2%) | | 5 (1.6%) |  | 4 (1.7%) | 5 (2.1%) |  |
| Marital status |  | |  | 0.769 |  |  | 0.690 |
| Unmarried | 128 (40.1%) | | 122 (38.4%) |  | 96 (40.2%) | 90 (37.7%) |  |
| Married | 172 (53.9%) | | 173 (54.4%) |  | 130 (54.4%) | 132 (55.2%) |  |
| Unknown | 19 (6.0%) | | 23 (7.2%) |  | 13 (5.4%) | 17 (7.1%) |  |
| Grade |  | |  | 0.585 |  |  | 0.704 |
| Well-moderate | 97 (30.4%) | | 85 (26.7%) |  | 73 (30.5%) | 67 (28.0%) |  |
| Poor-undifferentiated | 74 (23.2%) | | 79 (24.8%) |  | 52 (21.8%) | 59 (24.7%) |  |
| Unknown/other | 148 (46.4%) | | 154 (48.5%) |  | 114 (47.7%) | 113 (47.3%) |  |
| Tumor size |  | |  | 0.770 |  |  | 0.264 |
| ≤3.0cm | 73 (22.9%) | | 75 (23.6%) |  | 47 (19.7%) | 63 (26.4%) |  |
| 3.0-5.0cm | 36 (11.3%) | | 37 (11.6%) |  | 24 (10.0%) | 28 (11.7%) |  |
| >5.0cm | 14 (4.4%) | | 9 (2.8%) |  | 9 (3.8%) | 9 (3.8%) |  |
| unknown | 196 (61.4%) | | 197 (62.0%) |  | 159 (66.5%) | 139 (58.2%) |  |
| Extension |  | |  | 0.861 |  |  | 0.119 |
| E1 | 74 (23.2%) | | 84 (26.4%) |  | 43 (18.0%) | 62 (25.9%) |  |
| E2 | 34 (10.7%) | | 35 (11.0%) |  | 22 (9.2%) | 27 (11.3%) |  |
| E3 | 76 (23.8%) | | 67 (21.1%) |  | 49 (20.5%) | 52 (21.8%) |  |
| E4 | 7 (2.2%) | | 6 (1.9%) |  | 7 (2.9%) | 5 (2.1%) |  |
| Ex | 128 (40.1%) | | 126 (39.6%) |  | 118 (49.4%) | 93 (38.9%) |  |
| N classification |  | |  | 0.815 |  |  | 0.081 |
| N0 | 130 (40.8%) | | 137 (43.1%) |  | 88 (36.8%) | 103 (43.1%) |  |
| N1 | 44 (13.8%) | | 44 (13.8%) |  | 28 (11.7%) | 37 (15.5%) |  |
| Nx | 145 (45.4%) | | 137 (43.1%) |  | 123 (51.5%) | 99 (41.4%) |  |
| M classification |  | |  | 0.137 |  |  | 0.076 |
| M0 | 171 (53.6%) | | 161 (50.6%) |  | 108 (45.2%) | 130 (54.4%) |  |
| M1 | 4 (1.3%) | | 1 (0.3%) |  | 3 (1.3%) | 1 (0.4%) |  |
| M2 | 15 (4.7%) | | 27 (8.5%) |  | 13 (5.4%) | 18 (7.5%) |  |
| Mx | 129 (40.4%) | | 129 (40.6%) |  | 115 (48.1%) | 90 (37.7%) |  |
| Histology |  | |  | <0.001 |  |  | 0.019 |
| SCC | 157 (49.2%) | | 180 (56.6%) |  | 157 (49.2%) | 180 (56.6%) |  |
| SGC | 91 (28.5%) | | 45 (14.2%) |  | 91 (28.5%) | 45 (14.2%) |  |
| Other/unknown | 71 (21.3%) | | 93 (29.2%) |  | 71 (22.3%) | 93 (29.2%) |  |

PSM: propensity-score matching, SCC: squamous cell carcinoma, SGC: salivary gland-type carcinoma
