# Supplementary material for: The impact of surgery and age on mortality with primary trachea malignant tumors: a retrospective study based on propensity-score matching analysis
Source: J Cardiothorac Surg. 2023 Jul 10;18:224. doi: 10.1186/s13019-023-02340-z (PMC10334644; doi:10.1186/s13019-023-02340-z)
Supplement: Supplementary file 2 — Additional File 2: The clinical and pathological characteristics before and after PSM according to the surgery group. [file 13019_2023_2340_MOESM2_ESM.docx]

**Supplementary table 2**: The clinical and pathological characteristics before and after PSM according to the surgery group.

|  | | **Surgery** | |  | **Surgery (PSM)** | |  |
| --- | --- | --- | --- | --- | --- | --- | --- |
| **Variables** | **No** | | **Yes** | ***P* value** | **No** | **Yes** | ***P* value** |
|  | **N=474** | | **N=127** |  | **N=111** | **N=111** |  |
| Race |  | |  | 0.296 |  |  | 0.645 |
| Caucasians | 375 (79.1%) | | 95 (74.8%) |  | 81 (73.0%) | 84 (75.7%) |  |
| Other | 99 (20.9%) | | 32 (25.2%) |  | 30 (27.0%) | 27 (24.3%) |  |
| Sex |  | |  | 0.076 |  |  | 0.893 |
| Male | 284 (59.9%) | | 65 (51.2%) |  | 61 (55.0%) | 60 (54.1%) |  |
| Female | 190 (40.1%) | | 62 (48.8%) |  | 50 (45.0%) | 51 (45.9%) |  |
| Age (year) |  | |  | <0.001 |  |  | 0.680 |
| <65 | 212 (44.7%) | | 84 (66.1%) |  | 66 (59.5%) | 69 (62.2%) |  |
| >64 | 262 (55.3%) | | 43 (33.9%) |  | 45 (40.5%) | 42 (37.8%) |  |
| Chemotherapy |  | |  | <0.001 |  |  | 0.456 |
| No | 275 (58.0%) | | 108 (85.0%) |  | 96 (86.5%) | 92 (82.9%) |  |
| Yes | 199 (42.0%) | | 19 (15.0%) |  | 15 (13.5%) | 19 (17.1%) |  |
| Radiotherapy |  | |  | 0.994 |  |  | 0.568 |
| No | 157 (33.1%) | | 42 (33.1%) |  | 43 (38.7%) | 39 (35.1%) |  |
| Yes | 305 (64.3%) | | 82 (64.6%) |  | 64 (57.7%) | 70 (63.1%) |  |
| Unknown | 12 (2.6%) | | 3 (2.3%) |  | 4 (3.6%) | 2 (1.8%) |  |
| Marital status |  | |  | 0.091 |  |  | 0.073 |
| Unmarried | 195 (41.1%) | | 40 (31.5%) |  | 42 (37.8%) | 37 (33.3%) |  |
| Married | 247 (52.1%) | | 80 (63.0%) |  | 56 (50.5%) | 69 (62.2%) |  |
| Unknown | 32 (6.8%) | | 23 (5.5%) |  | 13 (11.7%) | 5 (4.5%) |  |
| Grade |  | |  | 0.025 |  |  | 0.376 |
| Well-moderate | 127 (26.8%) | | 45 (35.4%) |  | 41 (36.9%) | 37 (33.3%) |  |
| Poor-undifferentiated | 112 (23.6%) | | 36 (28.3%) |  | 23 (20.7%) | 32 (28.8%) |  |
| Unknown/other | 235 (49.6%) | | 46 (36.3%) |  | 47 (42.4%) | 42 (37.9%) |  |
| Tumor size |  | |  | <0.001 |  |  | 0.002 |
| ≤3.0cm | 84 (17.7%) | | 60 (47.2%) |  | 26 (23.4%) | 47 (42.3%) |  |
| 3.0-5.0cm | 59 (12.4%) | | 10 (7.9%) |  | 12 (10.8%) | 8 (7.2%) |  |
| >5.0cm | 18 (3.8%) | | 4 (3.1%) |  | 0 (0.0%) | 4 (3.6%) |  |
| unknown | 313 (66.1%) | | 53 (41.8%) |  | 73 (65.8%) | 52 (46.9%) |  |
| Extension |  | |  | 0.150 |  |  | <0.001 |
| E1 | 114 (24.1%) | | 39 (30.7%) |  | 9 (8.1%) | 35 (31.5%) |  |
| E2 | 60 (12.7%) | | 7 (5.5%) |  | 11 (9.9%) | 7 (6.3%) |  |
| E3 | 107 (22.6%) | | 31 (24.4%) |  | 11 (9.9%) | 21 (18.9%) |  |
| E4 | 11 (2.3%) | | 2 (1.6%) |  | 2 (1.8%) | 1 (0.9%) |  |
| Ex | 182 (38.3%) | | 48 (37.8%) |  | 78 (70.3%) | 47 (42.4%) |  |
| N classification |  | |  | 0.005 |  |  | <0.001 |
| N0 | 193 (40.7%) | | 68 (53.5%) |  | 26 (23.4%) | 56 (50.5%) |  |
| N1 | 75 (15.8%) | | 8 (6.3%) |  | 13 (11.7%) | 5 (4.5%) |  |
| Nx | 206 (43.5%) | | 51 (40.2%) |  | 72 (64.9%) | 50 (45.0%) |  |
| M classification |  | |  | 0.025 |  |  | 0.002 |
| M0 | 245 (51.7%) | | 76 (59.8%) |  | 35 (31.5%) | 62 (55.9%) |  |
| M1 | 5 (1.1%) | | 0 (0.0%) |  | 1 (0.9%) | 0 (0.0%) |  |
| M2 | 40 (8.4%) | | 2 (1.6%) |  | 7 (6.3%) | 2 (1.8%) |  |
| Mx | 184 (38.8%) | | 49 (38.6%) |  | 68 (61.3%) | 47 (42.3%) |  |
| Histology |  | |  | <0.001 |  |  | 0.004 |
| SCC | 276 (58.2%) | | 41 (32.3%) |  | 56 (50.5%) | 180 (33.3%) |  |
| SGC | 66 (13.9%) | | 60 (47.2%) |  | 26 (23.4%) | 45 (44.1%) |  |
| Other/unknown | 132 (27.9%) | | 26 (20.5%) |  | 29 (26.1%) | 93 (22.6%) |  |

PSM: propensity-score matching, SCC: squamous cell carcinoma, SGC: salivary gland-type carcinoma
